# Causal Evaluation of Laboratory Markers in Type 2 Diabetes on Cancer and Vascular Diseases Using Various Mendelian Randomization Tools

**DOI:** 10.3389/fgene.2020.597420

**Published:** 2020-12-21

**Authors:** Heejin Jin, Sanghun Lee, Sungho Won

**Affiliations:** ^1^Department of Public Health Sciences, Seoul National University, Seoul, South Korea; ^2^Department of Biostatistics, Medical Research Collaborating Center, Seoul National University Boramae Hospital, Seoul, South Korea; ^3^Department of Medical Consilience, Graduate School, Dankook University, Yongin-si, South Korea; ^4^Institute of Health and Environment, Seoul National University, Seoul, South Korea; ^5^RexSoft Corp., Seoul, South Korea

**Keywords:** mendelian randomization, instrumental variables analysis, causal relationship, risk factor, fasting glucose, 2-h postload glucose, glycated hemoglobin A1c

## Abstract

Multiple studies have demonstrated the effects of type 2 diabetes (T2D) on various human diseases; however, most of these were observational epidemiological studies that suffered from many potential biases including reported confounding and reverse causations. In this article, we investigated whether cancer and vascular disease can be affected by T2D-related traits, including fasting plasma glucose (FPG), 2-h postprandial glucose (2h-PG), and glycated hemoglobin A1c (HbA1c) levels, by using Mendelian randomization (MR). The summary statistics for FPG, 2h-PG, and HbA1c level were obtained through meta-analyses of large-scale genome-wide association studies that included data from 133,010 nondiabetic individuals from collaborating Meta-analysis of Glucose and Insulin Related Traits Consortium studies. Thereafter, based on the statistical assumptions for MR analyses, the most reliable approaches including inverse-variance-weighted (IVW), MR-Egger, MR-Egger with a simulation extrapolation (SIMEX), weighted median, and MR-pleiotropy residual sum and outlier (MR-PRESSO) methods were applied to identify traits affected by FPG, 2h-PG, and HbAlc. We found that coronary artery disease is affected by FPG, as per the IVW [log odds ratio (logOR): 0.21; *P* = 0.012], MR-Egger (SIMEX) (logOR: 0.22; *P* = 0.014), MR-PRESSO (logOR: 0.18; *P* = 0.045), and weighted median (logOR: 0.29; *P* < 0.001) methods but not as per the MR-Egger (logOR: 0.13; *P* = 0.426) approach. Furthermore, low-density lipoprotein cholesterol levels are affected by HbA1c, as per the IVW [beta (B): 0.23; *P* = 0.015), MR-Egger (B: 0.45; *P* = 0.046), MR-Egger (SIMEX) (B: 0.27; *P* = 0.007), MR-PRESSO (B; 0.14; *P* = 0.010), and the weighted median (B: 0.15; *P* = 0.012] methods. Further studies of the associated biological mechanisms are required to validate and understand the disease-specific differences identified in the TD2-related causal effects of each trait.

## Introduction

Type 2 diabetes (T2D) is characterized by high blood sugar, insulin resistance, and a relative lack of insulin and represents a common metabolic disorder worldwide. In its early stage, T2D is easy to ignore due to the lack of symptoms; however, chronic or poorly controlled T2D leads to eventually disabling or life-threatening complications. Numerous epidemiological studies have consistently demonstrated increased risks of cancer, vascular disease, nerve damage, and poor health-related outcomes in T2D patients (De Vegt et al., 1999; Laakso, 1999; Tsilidis et al., 2015), resulting in a shorter life expectancy (Collaboration, 2011). The main T2D-related complications reported in large-scale epidemiological studies tend to be malignant solid tumors (Johnson et al., 2012) and cardiovascular disease, including ischemic heart disease and stroke (Nesto, 2001; Bax et al., 2007; Gleissner et al., 2007; Young et al., 2009). However, the causal relationship between T2D and diverse health-related outcomes needs to be investigated and compared with the existing results.

Fasting plasma glucose (FPG) levels ≥126 mg/dl or postchallenge 2-h plasma glucose (2h-PG) levels ≥200 mg/dl in a 75-g 2-h oral glucose tolerance test (2h-OGTT) have been used as diagnostic criteria for T2D. Additionally, hemoglobin A1c (HbA1c) levels ≥6.5% were added to these diagnostic criteria in 2010 (Gavin Iii et al., 1997; Association, 2010). The three tests (FPG, 2h-PG, and HbA1c) are dependent on blood glucose metabolism status. Specifically, FPG assesses the state of stable sugar levels in the body following a temporary increase in externally administered sugar. The 2h-OGTT indicates how efficiently insulin is processed during metabolism in response to increased externally administered glucose. HbA1c reflects the average blood sugar level until immediately before the test and not at the time of sample collection because hemoglobin increases with time and according to glucose concentration (Nathan et al., 2007, 2008). A previous prospective cohort study demonstrates that the ability of the glycemic measures (FPG, 2h-PG, and HbA1c) to predict all-cause and cardiovascular mortality is different (Reddigan et al., 2010). Therefore, it is necessary to investigate the causal effects of these three T2D-related traits in the blood and how they differ in subsequent pathological disorders.

To efficiently identify causal associations between T2D-related traits and various phenotypes without potential biases or confounding and/or reverse causations, two-sample Mendelian randomization (MR) can be used to assess how genetic variants act as instruments for instrumental variable (IV) analysis aimed at estimating the causal effect of one trait on another. The two-sample MR refers to the fact that the associations of IV exposure and IV outcome were measured from two different samples (as opposed to one-sample MR). The two-sample MR is generally preferred and compared to a one-sample MR; a two-sample MR will not lead to inflated type 1 error rated and false-positive findings. Using genetic variants as instruments, which are not associated with conventional confounders of observational studies, allows the MR approach to be considered analogous to randomized controlled trials (Burgess and Thompson, 2015). MR analysis requires three assumptions: (1) IVs are strongly associated with intermediate exposure, (2) IVs are independent of confounders, and (3) IVs affect outcomes only through the exposure path (i.e., no directional horizontal pleiotropy effect). “Directional” horizontal pleiotropy indicates that the mean value of the pleiotropy distribution is nonzero. If these assumptions hold, an inverse-variance-weighted (IVW) method provides the most efficient and unbiased estimates of causal effects (Burgess et al., 2020). Various MR methods have been proposed for providing a more robust approach under weaker assumptions (Burgess et al., 2013; Bowden et al., 2015, 2016a,b; Verbanck et al., 2018).

The aim of this study was to assess the causal effect of T2D-related traits (FPG, 2h-PG, and HbA1c) on cancers and vascular diseases via MR analysis using several methods, including those measuring sensitive to assumption violations in the MR-Base platform database (Hemani et al., 2016).

## Materials and Methods

### Exposure Datasets

The exposure traits of interest were FPG, 2h-PG, and HbA1c. The summary statistics for T2D-related traits were obtained through large-scale genome-wide association study (GWAS) meta-analyses of 133,010 nondiabetic individuals from collaborating studies within the Meta-analysis of Glucose and Insulin Related Traits Consortium (MAGIC) (Scott et al., 2012). In most of these studies, participants were of European ancestry and were adults. A total of ~2.5 million genome-wide directly genotyped or imputed autosomal single-nucleotide polymorphisms (SNPs) were reported, including 36, 9, and 11 SNPs with genome-wide significant (*P* < 5 × 10^−8^) associations with FPG, 2h-PG, and HbA1c, explaining 4.8, 1.7, and 2.4% of the variance in the trait, respectively. Among these, SNPs were selected separately for each trait as IV candidates not in linkage disequilibrium (LD; *r*^2^ < 0.001) or within 10,000 kb of an established signal with exposures. To specify final IV sets, available genetic instruments for assessing outcome traits of interest were explored via the MR-Base platform database (https://www.mrbase.org/) whose registry comprises GWAS summary data including over 11 billion genetic variants related with various phenotypes from 1,673 GWAS or through the R package “TwoSampleMR” (https://rdrr.io/github/MRCIEU/TwoSampleMR/). To reflect the same reference strand between exposure and outcome, alleles and effects were harmonized using effect/noneffect alleles and minor allele frequency for palindromic SNPs.

### Outcome Datasets

Human phenotypes were divided into two categories of diseases or traits known to be related to T2D. The first category was cancer at major sites: breast, gall bladder, lung [adenocarcinoma and squamous cell (SC) carcinoma], ovarian, pancreatic, and thyroid (differentiated types). The second category was vascular disease: coronary kidney disease (CKD), coronary artery disease (CAD), stroke, cardioembolic stroke, small-vessel stroke, and high-density lipoprotein (HDL)/low-density lipoprotein (LDL) cholesterol levels. We obtained summary SNP-outcome associations with a total of 14 human health phenotypes through the MR-Base platform. Additionally, information regarding each outcome trait of interest was extracted (e.g., author/study/consortium name, number of cases and controls, publication year, PubMed ID, study population, unit, etc.) and listed in Table 1.

**Table 1:** Description of data from MR-Base based on the phenotype.

**Category**	**Trait**	**Consortium/First author**	**PubMed ID**	**Unit**	**No. of cases**	**No. of controls**	**No. of SNPs**	**Population**
**CANCER**								
	Breast cancer	BCAC	25,751,625	LogOR	15,748	18,084	13,011,123	European
	Lung cancer	ILCCO	24,880,342	LogOR	11,348	15,861	8,945,893	European
	Lung cancer (SC)	ILCCO	24,880,342	LogOR	3,275	150,038	8,893,750	European
	Ovarian cancer	OCAC	28,346,442	LogOR	1,366	40,941	11,403,952	European
	Pancreatic cancer	PanScan1	19,648,918	LogOR	1,896	1,939	521,863	European
	Thyroid cancer	Kohler A	23,894,154	LogOR	649	431	572,028	European
**VASCULAR DISEASE**								
	CAD	VanderHarst P	29,212,778	LogOR	122,733	424,528	7,934,254	European
	CAD^*^	UK Biobank	29,212,778	LogOR	34,541	261,984	7,904,237	European
	CKD	CKDGen	26,831,199	LogOR	12,385	104,780	2,191,877	Mixed
	HDL cholesterol	GLGC	24,097,068	mg/dL	–	187,167	2,447,442	Mixed
	LDL cholesterol	GLGC	24,097,068	mg/dL	–	173,082	2,437,752	Mixed
	LDL cholesterol^*^	Prins BP	2,8887,542	mg/dL	–	9,961	23,165,055	European
	Stroke	Malik R	29,531,354	LogOR	40,585	406,111	7,633,440	European
	(Cardio-embolic)	Malik R	29,531,354	LogOR	7,193	406,111	8,271,294	European
	(Small-vessel)	Malik R	29,531,354	LogOR	5,386	192,662	6,150,261	European

*SC, squamous cell; SD, standard deviation; CKD, chronic kidney disease; CAD, coronary artery disease; LDL, low-density lipoprotein; HDL, high-density lipoprotein; logOR, log odds ratio*.

* *Replication datasets*.

### MR Assumptions

**Figure 1:**
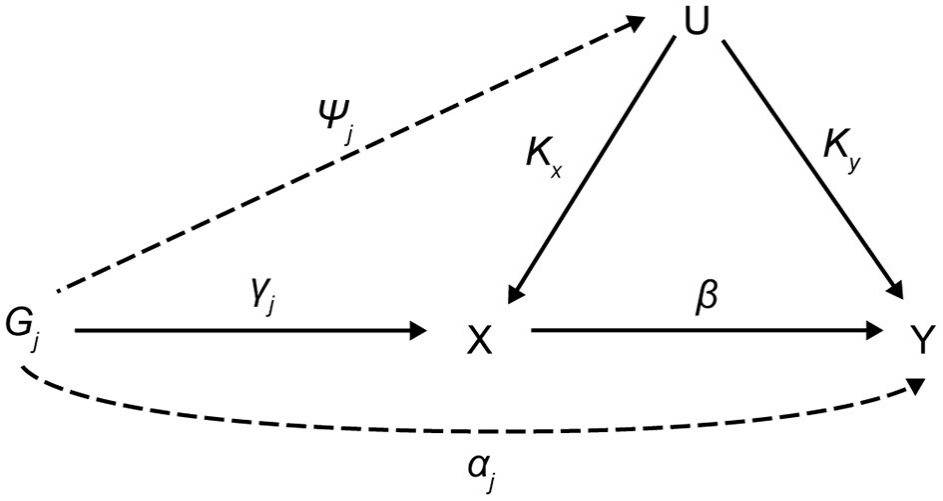
Causal directed acyclic graph for MR analysis. MR, Mendalian randomization; *G*_*j*_ (j = 1, 2, …, J), the genetic variant; X, exposure; Y, outcome; U, confounding factor; γ_j_, the association between *G*_*j*_ and X; α_*j*_, the association between *G*_*j*_ and Y; ψ_j_, associations between a U and *G*_*j*_; *K*_*x*_, associations between a *U* and X; *K*_*y*_, associations between a U and Y; β, causal estimate.

The assumptions of MR studies can be represented using causal directed acyclic graphs (DAGs) (Figure 1). In a DAG, the genetic variant *G*_*j*_ (*j* = 1, 2, …, *J*), and the exposure, *X*, are denoted as γ_*j*_, and the association between the genetic variant, *G*_*j*_, and the outcome, *Y*, is denoted as α_*j*_. Associations between a confounding factor (*U*) and *G*_*j*_, *X*, and *Y* are denoted as ψ_*j*_, *K*_*x*_, and *K*_*y*_, respectively. In a two-sample MR setting, we refer to $\hat{\gamma_{j}}$ as an estimate from the *j*th SNP-exposure association (with variance $\sigma_{X_{j}}^{2}$) from sample 1 and $\hat{\alpha_{j}}$ as an estimate from the *j*th SNP-outcome association (with variance $\sigma_{Y_{j}}^{2}$) from sample 2.

$$\begin{array}{lll} \text{Sample}1:\hat{\gamma_{j}} & = & \gamma_{j}+k_{x}\varphi_{j}+\epsilon_{X_{j}},\text{var}\left(\epsilon_{X_{j}}\right)=\;\sigma_{X_{j}}^{2}\\ \text{Sample}2:\hat{\alpha_{j}} & = & \alpha_{j}+k_{y}\varphi_{j}+\beta \left(\gamma_{j}+k_{x}\varphi_{j}\right)+\epsilon_{Y_{j}},\text{ var}\left(\epsilon_{Y_{j}}\right)=\;\sigma_{Y_{j}}^{2} \end{array}$$

The genetic variant, *G*_*j*_, for valid IVs must satisfy the following three core assumptions: (i) IV1, γ_*j*_ ≠ 0; (ii) IV2, φ_*j*_ = 0; (iii) IV3, α_*j*_ = 0. Furthermore, a two-sample MR requires a “no measurement error” (NOME) assumption and an instrument strength independent of direct effect (InSIDE) assumption. The former means that the SNP-exposure associations are estimated without measurement error $\sigma_{X_{j}}^{2}=0$, and the latter assumes cov(α_*j*_, γ_*j*_) = 0. It is important to assess the instrument strength to prevent weak instrument bias on MR analysis. We evaluated weak instruments with mean *F* statistics, where the *F* > 10, a commonly used threshold to avoid bias (Burgess et al., 2013; Bowden et al., 2016b). The degree of violation of the NOME assumption was quantified using the previously reported *I*^2^ statistic (ranging 0–1) (Bowden et al., 2016b). Higher values of *I*^2^ indicate less regression dilution of the causal estimates (i.e., less underestimation of the causal estimates), and the value of *I*^2^ close to 1 means the observed association is closer to the true effect.

### MR Methods

Using all genetic variants, *G*_*j*_, that satisfy the three IV assumptions and the NOME and InSIDE assumptions, the causal effect of exposure on the outcome can be consistently estimated from the weighted mean of the ratio estimates (α_*j*_/γ_*j*_) using an IVW method (Burgess et al., 2013). The IVW estimate is the most efficient method when all genetic variants satisfy all three IV assumptions. Cochran's *Q* statistic was used to quantify heterogeneity (Greco et al., 2015; Bowden et al., 2017).

However, the estimate could be biased if one or more variants are invalid. The weighted median method provides valid causal estimates, even if up to 50% of the instruments are invalid. The median is unaffected by outliers, making the weighted median estimate insensitive to a pleiotropic genetic variant. Causal effects are obtained from the weighted median of the ratio estimates in genetic instruments, resulting in smaller standard errors receiving more weight (Bowden et al., 2016a).

The MR-Egger method allows all SNPs to be used as invalid instruments but requires variants to satisfy the InSIDE assumption, enabling estimation of appropriate causal effects in the presence of pleiotropic effects (Bowden et al., 2015). This model is suitable for linear regression, and the intercept term, β_0*E*_, is interpreted as the average horizontal pleiotropic effect across the genetic variants (Bowden et al., 2015). Rücker's *Q*′ statistic from MR-Egger was used to quantify directional horizontal pleiotropy (Greco et al., 2015; Bowden et al., 2017). If estimates of β_0*E*_ equal to zero, the MR-Egger slope estimate will be the same as the IVW estimate (Burgess and Thompson, 2015). However, when the *I*^2^ statistic quantifying the strength of NOME violation for IVs for the MR-Egger method is low, a magnitude of regression dilution still occurs. In cases where the NOME assumption is violated, the SIMEX method can be used to correct attenuation bias (Bowden et al., 2016b).

Violation of IV3 (i.e., directional horizontal pleiotropy) can raise a severe bias in MR analysis. The MR-PRESSO test has an advantage over MR-Egger, in that it identifies and removes pleiotropic SNPs. The test comprises three parts: (1) the MR-PRESSO global test detects directional horizontal pleiotropy, (2) the outlier-corrected causal estimate corrects for the detected directional horizontal pleiotropy, and (3) the MR-PRESSO distortion test estimates whether the causal estimates differ significantly (*P* < 0.05) following adjustment for the outliers (Verbanck et al., 2018). Therefore, MR-PRESSO results are preferable in the presence of a horizontal pleiotropic effect.

The appropriate methods differ according to the assumptions satisfied, and the most suitable choices are presented in Table 2. The IVW method is the most efficient way to estimate the causal effect when all genetic variants are valid instruments (Burgess et al., 2020). In cases where the MR assumption of no pleiotropy is not met, the MR-PRESSO test detects possible outliers and provides consistent estimates following outlier removal (Burgess and Thompson, 2017). When some IVs are invalid (<50%) (majority of IVs do not exhibit directional horizontal pleiotropy), the weighted median approach can be used as an alternative method of providing a consistent estimate (Bowden et al., 2016a). We can check whether the assumptions for IV1–3 are satisfied for each SNPs through GWAS summary datasets (if *F* statistics is lower than 10, it is considered a violation of IV1 and if the *P* value for α_*j*_ is genome-wide significantly associated with outcomes, it is considered a violation of IV3). By contrast, MR-Egger can obtain a causal estimate by correcting directional horizontal pleiotropy but has the disadvantage of low power (Bowden et al., 2015). If the NOME assumption is violated (*I*^2^ < 90%), the MR-Egger (SIMEX) method would be suitable (Bowden et al., 2016b).

**Table 2:** Recommended MR methods by assumption of IVs.

**When no heterogeneity exists**						
**No weak IVs (*F* > 10)**	***Q^†^***	***Q^†^***	**RSS*^‡^***	**NOME** **(I^2^ > 90)**	**InSIDE**	**Recommended methods**
Satisfied				Satisfied	Satisfied	IVW
Violated				Satisfied	Violated	Weighted median
Satisfied				Violated	Satisfied	IVW
Violated				Violated	Satisfied	MR-Egger (SIMEX)
**When heterogeneity exists**						
**No weak IVs (*F* > 10)**	***Q***	***Q′***	**RSS**	**NOME** **(I^2^ > 90)**	**InSIDE**	**Recommended methods**
Satisfied	^✓^	^✓^		Satisfied	Satisfied	MR-Egger
		^✓^				IVW
	^✓^					MR-Egger
			^✓^			IVW
	^✓^	^✓^	^✓^			MR-PRESSO
		^✓^	^✓^			IVW
	^✓^		^✓^			MR-Egger, MR-PRESSO
Violated	^✓^	^✓^		Satisfied	Satisfied	MR-Egger
		^✓^				IVW
	^✓^					MR-Egger
			^✓^			MR-PRESSO
	^✓^	^✓^	^✓^			MR-PRESSO
		^✓^	^✓^			MR-PRESSO
	^✓^		^✓^			MR-Egger, MR-PRESSO
Satisfied	At least one violated			Violated	Satisfied	MR-Egger (SIMEX)
Violated	At least one violated			Violated	Satisfied	MR-Egger (SIMEX)

*IV, instrument variable; Q, P-value for the Q test from IVW; Q′, P-value for the Q′ test from MR-Egger; RSS, P-value for MR-PRESSO global test; NOME, no measurement error; InSIDE, instrument strength independent of direct effect; IVW, inverse-variance-weighted; SIMEX, simulation extrapolation; MR-PRESSO, MR-pleiotropy residual sum and outlier*.

✓ *, significant (heterogeneity exists)*.

† *Greco et al. (2015); Bowden et al. (2017)*.

‡ *Verbanck et al. (2018)*.

*The IVW estimate is the most efficient method when all genetic variants satisfy all three IV assumptions. The MR-Egger method allows all SNPs to be used as invalid instruments but requires variants to satisfy the InSIDE assumption, enabling estimation of appropriate causal effects in the presence of pleiotropic effect. However, when the I^2^ statistic quantifying the strength of NOME violation for IVs for the MR-Egger method is low, the SIMEX method can be used to correct attenuation bias. The MR-PRESSO test has an advantage over MR-Egger, in that it identifies and removes pleiotropic SNPs. The weighted median method provides valid causal estimates, even if up to 50% of the instruments are invalid. The median is unaffected by outliers, making the weighted median estimate insensitive to a pleiotropic genetic variant*.

### Bidirectional MR Analysis

We conducted bidirectional MR analysis to investigate the presence of reverse causality among associations between T2D-related traits and outcomes of interest. This was performed by switching the exposure and outcomes in opposite directions.

### MR Power Analysis

**Figure 2:**
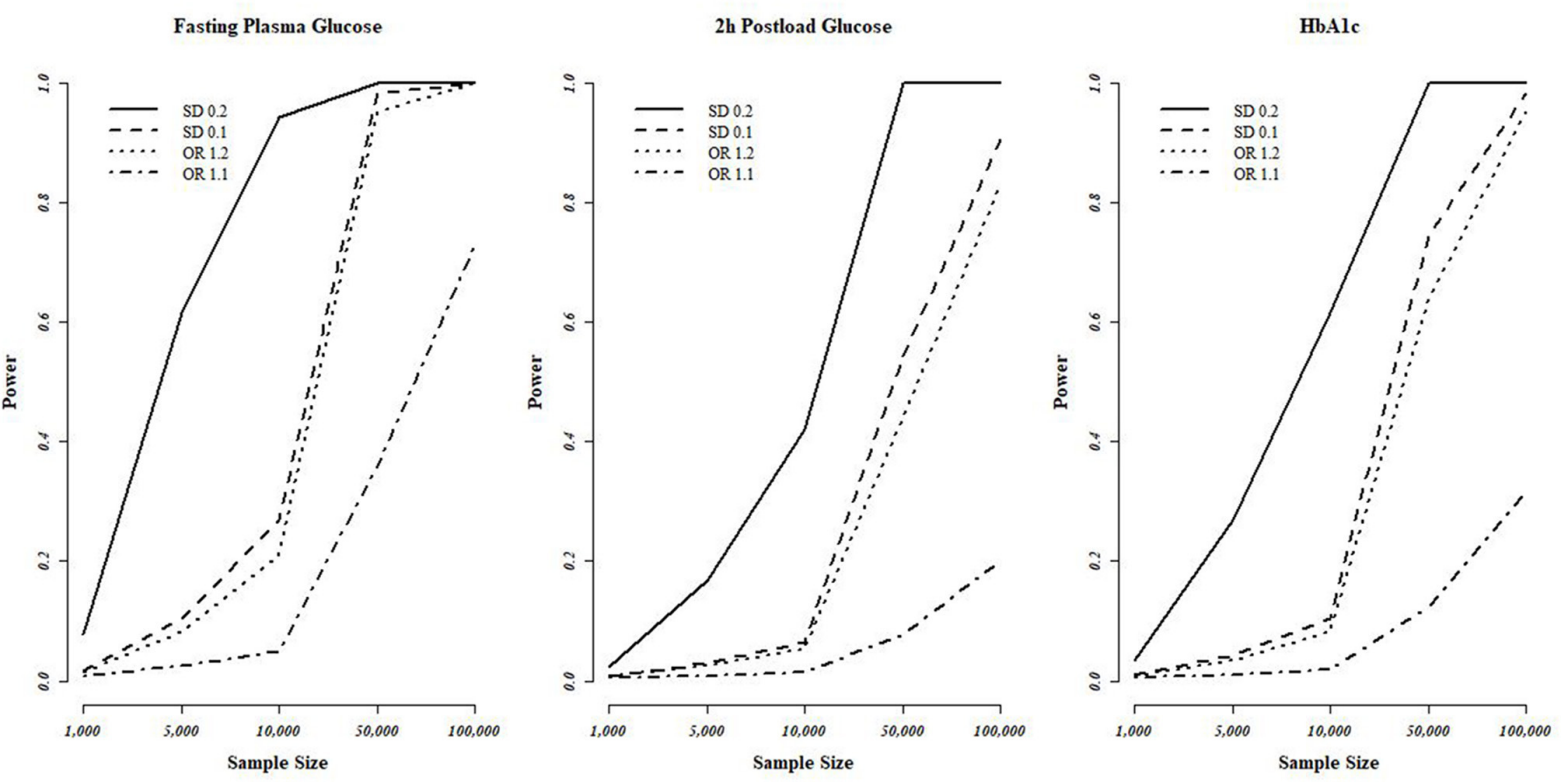
Statistical power evaluations of MR analyses based on the T2D-diagnosis criteria. FPG, 2h-PG, and HbA1c. We used a conservative significance threshold of *P* < 0.007 with Bonferroni correction using seven of testing. MR, Mendalian randomization; T2D, type 2 diabetes; FPG, fasting plasma glucose; 2h-PG, 2-h plasma glucose; HbA1c, hemoglobin A1c; OR, odds ratio; SD, standard deviation.

Power calculations were conducted at https://sb452.shinyapps.io/power/ (Burgess et al., 2020). The proportion of variance in the exposure explained by the genetic variants (*R*^2^) was required for MR power analysis, with 0.048 (FPG), 0.017 (2h-PG), and 0.024 (HbA1c) used, respectively. We assumed odds ratios (ORs) of 1.1 and 1.2 for binary outcomes and changes in outcomes in standard deviation (SD) units per SD change in exposure (0.1 and 0.2) for continuous outcomes. Statistical power evaluations at the conservative significance level [0.007 (Bonferroni correction with seven tests)] are plotted in Figure 2.

## Results

A total of 34, 7, and 11 genetic variants associated with FPG, 2h-PG, and HbA1c, respectively, were available as potential instruments from studies included in MAGIC. Each IV set showed genome-wide significant (*P* < 5 × 10^−8^) associations with T2D-related traits and were not in LD or within 10,000 kb of an established signal. To investigate IV quality, we generated F statistics, *I*^2^ values, and *P* values for Cochran's Q statistic from IVW, Rucker's *Q*′ statistic from MR-Egger, and MR-PRESSO global test (Table 3). All instruments used for MR analyses had *F* statistics > 10, indicating no evidence of weak instrument bias. Rejection of the null hypothesis of the Cochran's Q statistic for heterogeneity suggested potential pleiotropy in the genetic variants and did not indicate that the InSIDE assumptions were invalid. When pleiotropic effect was present, MR-Egger (with and without SIMEX) and MR-PRESSO were performed rather than using the IVW method. All IVs for FPG met the NOME assumptions, but IVs for HbA1c were only partially met and not at all for 2h-PG. When the NOME assumption was violated, the results of MR-Egger (SIMEX) were generated. Using these IVs, we performed MR analyses for a total of 13 human health phenotypes, with all results (3 exposures × 13 phenotypes × 5 methods = 195 results) presented in Table 4. The MR method we recommended is highlighted in bold letters. The application of Bonferroni correction to each disease category (0.05/6 = 0.008 for cancer and 0.05/7 = 0.007 for vascular disease) revealed two significant phenotypes (CAD and LDL level) associated with T2D-related traits. Additionally, we confirmed these relationships through bidirectional and replication analyses (Tables 5, 6).

**Table 3:** Assumption check for instrumental variables.

	**FPG (R^2^ = 0.048)**				**2h-PG (R^2^ = 0.017)**				**HbA1c (R^2^ =0.024)**				
**Outcome**	***N***	***F***	***I*^2^** **(%)**	***Q*** ***Q^′^***	***N***	***F***	***I*^2^** **(%)**	***Q*** ***Q^′^***	***N***	***F***	***I*^2^** **(%)**	***Q*** ***Q^′^***	**Recommended method**
				**RSS**				**RSS**				**RSS**	
**CANCER**													
Breast cancer	34	130.5	96.8	<0.001	7	43.5	29.8	<0.001	11	77.6	92.2	0.110	MR-PRESSO^†^
				<0.001				0.019				0.249	MR-Egger (SIMEX)^‡^
				<0.001				0.023				0.371	IVW^*^
Lung cancer	33	133.0	97.3	0.182	7	43.5	36.2	0.322	11	77.6	87.6	0.021	IVW^†^^,^^‡^^,^ ^*^
				0.183				0.397				0.063	
				0.211				0.258				0.089	
SC lung cancer	33	43.5	40.1	0.599	7	43.5	40.1	0.875	11	77.6	87.9	0.198	MR-Egger (SIMEX)^†^
				0.553				0.786				0.154	IVW^‡^^,^ ^*^
				0.477				0.913				0.216	
Ovarian cancer	33	133.5	97.4	0.530	7	43.5	28.1	0.323	11	77.6	87.8	0.243	IVW^†^^,^^‡^^,^ ^*^
				0.490				0.224				0.334	
				0.412				0.362				0.298	
Pancreatic cancer	25	111.7	96.4	0.138	6	45.0	31.8	0.710	8	82.1	90.9	0.239	IVW^†^^,^^‡^^,^ ^*^
				0.122				0.594				0.186	
				0.199				0.642				0.214	
Thyroid cancer	27	144.9	97.9	0.183	5	47.4	21.9	0.434	9	81.7	91.2	0.882	IVW^†^^,^^‡^^,^ ^*^
				0.248				0.703				0.899	
				0.275				0.621				0.885	
**CARDIOVASCULAR DISEASE**													
CAD	34	130.5	97.3	<0.001	7	43.5	23.0	<0.001	11	77.6	88.0	0.002	MR-PRESSO^†^
				<0.001				<0.001				0.014	MR-Egger (SIMEX)^‡^
				<0.001				<0.001				0.018	IVW^*^
CKD	32	135.8	97.4	0.106	7	43.5	29.1	0.482	10	82.1	86.5	0.632	IVW^†^^,^^‡^^,^ ^*^
				0.114				0.451				0.548	
				0.151				0.395				0.421	
HDL cholesterol	34	130.5	97.3	<0.001	7	43.5	2.4	<0.001	11	77.6	90.9	0.545	MR-PRESSO^†^
				<0.001				<0.001				0.589	MR-Egger (SIMEX)^‡^
				<0.001				<0.001				0.436	IVW^*^
LDL cholesterol	34	130.8	97.2	<0.001	7	43.5	5.7	<0.001	11	77.6	91.1	<0.001	MR-PRESSO^†^^,^ ^*^
				<0.001				<0.001				<0.001	MR-Egger (SIMEX)^‡^
				<0.001				<0.001				<0.001	
Stroke	34	130.5	97.3	<0.001	7	43.5	39.9	0.069	11	77.6	86.0	0.075	MR-PRESSO^†^
				<0.001				0.051				0.084	IVW^‡^^,^ ^*^
				<0.001				0.067				0.092	
(Cardio-embolic)	34	130.5	97.4	0.498	7	43.4	35.2	0.733	11	77.6	88.7	0.255	IVW^†^^,^^‡^^,^ ^*^
				0.649				0.611				0.247	
				0.557				0.694				0.275	
(Small-vessel)	32	135.8	97.6	0.038	7	43.5	55.6	0.675	10	76.88	86.6	0.556	MR-Egger^†^
				0.130				0.636				0.522	IVW^‡^^,^ ^*^
				0.199				0.645				0.512	

*N, number of instruments; F, F statistic; Q, P-value for the Q test from IVW; Q′, P-value for the Q′ test from MR-Egger; RSS, P-value for MR-PRESSO global test; SC, squamous cell; CKD, chronic kidney disease; CAD, coronary artery disease; LDL, low-density lipoprotein; HDL, high-density lipoprotein; FPG, fasting plasma*.

† *Recommended method when FPG used as exposure*.

‡ *Recommended method when 2h-PG used as exposure*.

* *Recommended method when HbA1c used as exposure*.

*F-tests the weak instruments bias, I^2^ tests the NOME assumptions and Q, Q′, and RSS test the heterogeneity*.

**Table 4:** MR results for T2D-related traits on cancers and vascular disease.

		**FPG**			**2h-PG**			**HbA1c**		
**Trait and** **MR methods**	**Parameter**	***N***	**Estimate** **(95% CI)**	***P***	***N***	**Estimate (95% CI)**	***P***	***N***	**Estimate** **(95% CI)**	***P***
**CANCER**										
**BREAST CANCER**										
IVW	Estimate	34	−0.05 (−0.28, 0.18)	0.671	7	0.16 (−0.06, 0.37)	0.151	11	**−0.01 (−0.28, 0.25)**	**0.922**
MR-Egger	Intercept		0.00 (−0.01, 0.01)	0.537		−0.09 (−0.16, −0.01)	0.028		0.02 (0.00, 0.04)	0.067
	Slope		−0.18 (−0.65, 0.29)	0.456		1.08 (0.24, 1.92)	0.012		−0.46 (−0.98, 0.07)	0.092
MR-Egger (SIMEX)	Intercept		0.00 (−0.01, 0.01)	0.996		**0.00 (−0.02, 0.03)**	**0.941**		0.01 (−0.01, 0.02)	0.301
	Slope		−0.05 (−0.29, 0.19)	0.679		**0.15 (−0.11, 0.42)**	**0.303**		−0.04 (−0.30, 0.23)	0.800
MR-PRESSO	Estimate	33	**−0.10 (−0.32, 0.36)**	**0.364**	5	0.16 (−0.01, 0.34)	0.068		No outlier	–
Weighted median	Estimate		−0.20 (−0.44, 0.04)	0.094		0.21 (0.06, 0.36)	0.008		−0.03 (−0.32, 0.29)	0.842
**LUNG CANCER**										
IVW	Estimate	33	**0.05 (−0.22, 0.32)**	**0.721**	7	**−0.13 (−0.32, 0.06)**	**0.188**	11	**0.36 (−0.16, 0.88)**	**0.173**
MR-Egger	Intercept		0.01 (−0.01, 0.02)	0.330		−0.06 (−0.15, 0.03)	0.182		0.04 (−0.01, 0.08)	0.102
	Slope		−0.17 (−0.68, 0.35)	0.523		0.48 (−0.43, 1.39)	0.301		−0.50 (−1.65, 0.64)	0.389
MR-Egger (SIMEX)	Intercept		−0.01 (−0.02, 0.01)	0.148		0.00 (−0.02, 0.03)	0.795		0.00 (−0.02, 0.02)	0.898
	Slope		0.14 (−0.19, 0.35)	0.351		−0.15 (−0.39, 0.09)	0.286		0.37 (−0.19, 0.94)	0.233
MR-PRESSO	Estimate		No outlier	–		No outlier	–		No outlier	–
Weighted median	Estimate		−0.03 (−0.37, 0.32)	0.880		−0.08 (−0.32, 0.16)	0.494		0.04 (−0.47, 0.54)	0.886
**SC LUNG CANCER**										
IVW	Estimate	33	0.39 (0.02, 0.76)	0.037	7	**−0.19 (−0.46, 0.08)**	**0.161**	11	**0.51 (−0.12, 1.15)**	**0.115**
MR-Egger	Intercept		0.00 (−0.02, 0.02)	0.792		0.00 (−0.13, 0.13)	0.968		0.01 (−0.05, 0.07)	0.651
	Slope		0.31 (−0.38, 1.00)	0.376		−0.17 (−1.52, 1.19)	0.811		0.18 (−1.38, 1.75)	0.818
MR-Egger (SIMEX)	Intercept		**0.00 (−0.01, 0.01)**	**0.480**		0.01 (−0.01, 0.03)	0.307		−0.01 (−0.04, 0.02)	0.453
	Slope		**0.41 (0.05, 0.77)**	**0.032**		−0.26 (−0.46, −0.06)	0.052		0.58 (−0.09, 1.26)	0.127
MR-PRESSO	Estimate		No outlier	–		No outlier	–		No outlier	–
Weighted median	Estimate		0.36 (−0.17, 0.88)	0.180		−0.16 (−0.50, 0.17)	0.341		0.27 (−0.49, 1.03)	0.493
**OVARIAN CANCER**										
IVW	Estimate	33	**−0.14 (−0.68, 0.42)**	**0.632**	7	**0.14 (−0.29, 0.58)**	**0.526**	11	**0.16 (−0.73, 1.06)**	**0.719**
MR-Egger	Intercept		0.08 (−0.96, 1.13)	0.882		−0.01 (−0.24, 0.21)	0.905		0.06 (−0.02, 0.13)	0.142
	Slope		−0.01 (−0.04, 0.02)	0.637		0.28 (−2.12, 2.69)	0.817		−1.19 (−3.18, 0.80)	0.242
MR-Egger (SIMEX)	Intercept		−0.01 (−0.02, 0.01)	0.426		−0.04 (−0.07, −0.01)	0.044		0.03 (0.00, 0.06)	0.089
	Slope		−0.11 (−0.66, 0.44)	0.705		0.39 (0.03, 0.75)	0.087		−0.03 (−0.85, 0.79)	0.948
MR-PRESSO	Estimate		No outlier	–		No outlier	–		No outlier	–
Weighted median	Estimate		−0.46 (−1.33, 0.40)	0.293		−0.01 (−0.54, 0.53)	0.980		0.08 (−1.05, 1.21)	0.886
**PANCREATIC CANCER**										
IVW	Estimate	25	**−0.14 (−1.07, 0.79)**	**0.768**	6	**−0.01 (−0.50, 0.49)**	**0.973**	8	**0.53 (−0.64, 1.71)**	**0.374**
MR-Egger	Intercept		0.02 (−0.03, 0.06)	0.537		−0.04 (−0.27, 0.18)	0.702		0.03 (−0.08, 0.14)	0.592
	Slope		−0.64 (−2.49, 1.21)	0.497		0.45 (−1.94, 2.84)	0.713		−0.11 (−2.78, 2.56)	0.934
MR-Egger (SIMEX)	Intercept		−0.01 (−0.04, 0.02)	0.455		0.00 (−0.01, 0.05)	0.874		0.01 (−0.04, 0.06)	0.792
	Slope		−0.07 (−1.03, 0.89)	0.883		0.02 (−0.53, 0.57)	0.942		0.51 (−0.77, 1.79)	0.464
MR-PRESSO	Estimate		No outlier	–		No outlier	–		No outlier	–
Weighted median	Estimate		−0.81 (−2.01, 0.38)	0.184		−0.05 (−0.67, 0.57)	0.883		0.46 (−0.87, 1.78)	0.500
**THYROID CANCER**										
IVW	Estimate	27	**−0.34 (−1.67, 0.98)**	**0.612**	5	**−0.53 (−1.43, 0.38)**	**0.257**	9	**0.68 (−0.97, 2.33)**	**0.417**
MR-Egger	Intercept		0.06 (−0.01, 0.13)	0.116		0.32 (−0.08, 0.73)	0.122		−0.07 (−0.21, 0.07)	0.349
	Slope		−1.89 (−4.22, 0.43)	0.110		−3.97 (−8.43, 0.49)	0.081		2.30 (−1.47, 6.07)	0.231
MR-Egger (SIMEX)	Intercept		0.00 (−0.05, 0.04)	0.918		0.06 (−0.04, 0.17)	0.333		−0.02 (−0.07, 0.02)	0.344
	Slope		−0.34 (−1.73, 1.05)	0.641		−0.98 (−2.12, 0.16)	0.192		0.89 (−0.30, 2.09)	0.186
MR-PRESSO	Estimate		No outlier	–		No outlier	–		No outlier	–
Weighted median	Estimate		−1.14 (−2.73, 0.44)	0.157		−0.80 (−1.98, 0.38)	0.184		1.19 (−0.89, 3.28)	0.262
**CARDIOVASCULAR DISEASE**										
**CAD**										
IVW	Estimate	34	0.21 (0.05, 0.37)	0.012	7	0.12 (−0.06, 0.31)	0.183	11	0.24 (0.02, 0.46)	0.031
MR-Egger	Intercept		0.00 (−0.01, 0.01)	0.530		0.05 (−0.03, 0.14)	0.231		0.02 (−0.01, 0.03)	0.078
	Slope		0.13 (−0.18, 0.43)	0.426		−0.42 (−1.32, 0.48)	0.365		−0.14 (−0.60, 0.33)	0.569
MR-Egger (SIMEX)	Intercept		0.00 (−0.01, 0.01)	0.443		**0.00 (−0.02, 0.02)**	**0.833**		0.00 (−0.01, 0.01)	0.762
	Slope		0.22 (0.06, 0.39)	0.014		**0.14 (−0.09, 0.37)**	**0.301**		0.23 (−0.01, 0.47)	0.085
MR-PRESSO	Estimate	32	**0.18 (0.01, 0.35)**	**0.045**	5	0.21 (0.13, 0.29)	<0.001	10	**0.19 (−0.01, 0.39)**	**0.069**
Weighted median	Estimate		0.29 (0.14, 0.45)	<0.001		0.21 (0.10, 0.31)	<0.001		0.09 (−0.11, 0.30)	0.369
**CKD**										
IVW	Estimate	32	**0.12(−0.13, 0.36)**	**0.351**	7	**0.08 (−0.07, 0.23)**	**0.301**	10	**0.16 (−0.16, 0.47)**	**0.337**
MR-Egger	Intercept		−0.01(−0.02, 0.01)	0.281		0.01 (−0.06, 0.09)	0.760		−0.01 (−0.03, 0.02)	0.697
	Slope		0.33(−0.13, 0.78)	0.159		−0.04 (−0.83, 0.75)	0.916		0.29 (−0.48, 1.07)	0.455
MR-Egger (SIMEX)	Intercept		0.01 (−0.01, 0.01)	0.109		0.01 (−0.01, 0.02)	0.555		0.00 (−0.01, 0.01)	0.785
	Slope		0.08 (−0.16, 0.32)	0.496		0.05 (−0.13, 0.23)	0.601		0.17 (−0.14, 0.49)	0.315
MR-PRESSO	Estimate		No outlier	–		No outlier	–		No outlier	–
Weighted median	Estimate		0.17(−0.12, 0.46)	0.252		0.09 (−0.10, 0.28)	0.359		0.24 (−0.17, 0.65)	0.243
**HDL CHOLESTEROL**										
IVW	Estimate	34	−0.08 (−0.08, 0.25)	0.320	7	0.07 (−0.08, 0.23)	0.342	11	**0.19 (−0.11, 0.51)**	**0.206**
MR-Egger	Intercept		0.01 (0.00, 0.02)	0.219		0.02 (−0.06, 0.09)	0.680		0.03 (0.00, 0.06)	0.049
	Slope		−0.08 (−0.39, 0.23)	0.606		−0.10 (−0.94, 0.74)	0.817		−0.37 (−1.01, 0.27)	0.252
MR-Egger (SIMEX)	Intercept		0.00 (−0.01, 0.01)	0.728		**−0.01 (−0.03, 0.00)**	**0.064**		0.01 (−0.01, 0.02)	0.227
	Slope		0.09 (−0.08, 0.26)	0.259		**0.15 (0.02, 0.28)**	**0.074**		0.17 (−0.12, 0.47)	0.282
MR-PRESSO	Estimate	28	**0.04 (−0.03, 0.09)**	**0.265**	5	0.04 (−0.02, 0.11)	0.206		No outlier	–
Weighted median	Estimate		0.03 (−0.05, 0.10)	0.514		0.01 (−0.05, 0.06)	0.833		0.07 (−0.32, 0.46)	0.729
**LDL CHOLESTEROL**										
IVW	Estimate	34	0.02 (−0.16, 0.20)	0.807	7	0.04 (−0.11, 0.19)	0.626	11	0.23 (0.05, 0.41)	0.015
MR-Egger	Intercept		0.01 (−0.01, 0.01)	0.394		0.02 (−0.05, 0.09)	0.566		−0.01 (−0.03, 0.01)	0.281
	Slope		−0.10 (−0.44, 0.24)	0.551		−0.19 (−1.00, 0.61)	0.636		0.45 (0.01, 0.88)	0.046
MR-Egger (SIMEX)	Intercept		0.00 (−0.01, 0.01)	0.341		**−0.01 (−0.03, 0.01)**	**0.143**		−0.01 (−0.02, 0.00)	0.053
	Slope		0.04 (−0.14, 0.22)	0.695		**0.10 (−0.05, 0.25)**	**0.241**		0.27 (0.11, 0.44)	0.007
MR-PRESSO	Estimate	27	**0.04 (−0.03, 0.11)**	**0.225**	5	0.06 (0.01, 0.11)	0.033	10	**0.14 (0.03, 0.25)**	**0.010**
Weighted median	Estimate		−0.01 (−0.08, 0.07)	0.890		0.04 (−0.02, 0.10)	0.214		0.15 (0.03, 0.26)	0.012
**STROKE**										
IVW	Estimate	34	0.15 (−0.03, 0.33)	0.104	7	**0.06 (−0.06, 0.17)**	**0.338**	11	**−0.06 (−0.27, 0.15)**	**0.567**
MR-Egger	Intercept		0.01 (0.00, 0.02)	0.005		−0.02 (−0.07, 0.04)	0.579		0.01 (−0.01, 0.03)	0.308
	Slope		−0.24 (−0.56, 0.08)	0.137		0.22 (−0.37, 0.81)	0.467		−0.30 (−0.81, 0.21)	0.244
MR-Egger (SIMEX)	Intercept		0.00 (−0.01, 0.01)	0.174		0.00 (−0.01, 0.01)	0.930		0.00 (−0.01, 0.01)	0.396
	Slope		0.17 (−0.01, 0.35)	0.078		0.06 (−0.09, 0.21)	0.469		−0.09 (−0.31, 0.13)	0.451
MR-PRESSO	Estimate	33	**0.13 (−0.04, 0.31)**	**0.135**		No outlier	–		No outlier	–
Weighted median	Estimate		0.02 (−0.16, 0.19)	0.835		0.04 (−0.08, 0.16)	0.506		−0.15 (−0.38, 0.08)	0.205
**STROKE (CARDIO-EMBOLIC)**										
IVW	Estimate	34	**0.22 (−0.05, 0.48)**	**0.118**	7	**0.06 (−0.13, 0.26)**	**0.530**	11	**−0.51 (−0.94**, **−0.04)**	**0.023**
MR-Egger	Intercept		0.01 (0.00, 0.03)	0.047		0.00 (−0.09, 0.09)	0.988		0.02 (−0.02, 0.06)	0.369
	Slope		−0.23 (−0.74, 0.29)	0.387		0.07 (−0.91, 1.05)	0.889		−0.93 (−1.96, 0.09)	0.076
MR-Egger (SIMEX)	Intercept		0.00 (−0.01, 0.01)	0.266		−0.01 (−0.02, 0.01)	0.383		0.01 (−0.01, 0.02)	0.403
	Slope		−0.03 (−0.03, 0.51)	0.094		0.11 (−0.07, 0.29)	0.294		−0.56 (−1.01, −0.10)	0.039
MR-PRESSO	Estimate		No outlier	–		No outlier	–		No outlier	–
Weighted median	Estimate		0.03 (−0.36, 0.41)	0.889		0.01 (−0.24, 0.25)	0.937		−0.55 (−1.09, −0.02)	0.045
**STROKE (SMALL-VESSEL)**										
IVW	Estimate	32	**0.34 (0.04, 0.64)**	**0.025**	7	**0.16 (−0.02, 0.33)**	**0.084**	10	**0.37 (0.01, 0.73)**	**0.046**
MR-Egger	Intercept		0.02 (0.01, 0.04)	0.016		0.03 (−0.05, 0.11)	0.443		−0.01 (−0.05, 0.02)	0.422
	Slope		−0.22 (−0.75, 0.32)	0.427		−0.16 (−1.00, 0.67)	0.700		0.71 (−0.19, 1.61)	0.124
MR-Egger (SIMEX)	Intercept		−0.01 (−0.01, 0.01)	0.209		0.00 (−0.02, 0.02)	0.744		0.00 (−0.02, 0.01)	0.551
	Slope		0.37 (0.08, 0.68)	0.020		0.11 (−0.07, 0.34)	0.255		0.42 (0.04, 0.79)	0.063
MR-PRESSO	Estimate		No outlier	–		No outlier	–		No outlier	–
Weighted median	Estimate		0.16 (−0.24, 0.55)	0.435		0.12 (−0.10, 0.35)	0.294		0.46 (−0.03, 0.94)	0.065

*MR, Mendalian randomization; N, number of instruments; T2D, type 2 diabetes; IVW, inverse-variance-weighted; SIMEX, simulation extrapolation; PRESSO (O-C), pleiotropy residual sum and outlier (outlier-correction); SC, squamous cell; LDL, low-density lipoprotein; HDL, high-density lipoprotein; CKD, chronic kidney disease; CAD, coronary artery disease; CI, confidence interval; FPG, fasting plasma glucose; 2h-PG, 2-h plasma glucose; Hb1Ac, hemoglobin A1c*.

**Table 5:** Significant results from MR and replication analyses.

					**Original study**			**Replication study**		
**No**.	**Exposure**	**Outcome**	**MR method**	**Parameter**	**N**	**Estimate (95% CI)**	***P***	***N***	**Estimate (95% CI)**	***P***
1	FPG	CAD	IVW	Estimate	34	0.21 (0.05, 0.37)	0.012	34	0.14 (−0.02, 0.29)	0.078
			MR Egger	Intercept		0.00 (−0.01, 0.01)	0.530		0.01 (0.00, 0.02)	0.090
				Slope		0.13 (−0.18, 0.43)	0.426		−0.07 (−0.36, 0.22)	0.626
			MR Egger (SIMEX)	Intercept		0.00 (−0.01, 0.01)	0.443		0.00 (−0.01, 0.01)	0.769
				Slope		0.22 (0.06, 0.39)	0.014		0.144 (−0.02, 0.30)	0.087
			MR-PRESSO (O-C)	Estimate	32	**0.18 (0.01, 0.35)**	**0.045**	28	**0.19 (0.07, 0.32)**	**0.002**
			Weighted median	Estimate		0.29 (0.14, 0.45)	<0.001		0.20 (0.07, 0.34)	0.003
2	HbA1c	LDL cholesterol	IVW	Estimate	11	0.23 (0.05, 0.41)	0.015	10	0.15 (−0.31, 0.61)	0.521
			MR Egger	Intercept		−0.01 (−0.03, 0.01)	0.281		−0.05 (−0.08, −0.01)	0.004
				Slope		0.45 (0.01, 0.88)	0.046		0.14 (0.39, 1.89)	0.003
			MR Egger (SIMEX)	Intercept		−0.01 (−0.02, 0.00)	0.053		0.00 (−0.02, 0.02)	0.780
				Slope		0.27 (0.11, 0.44)	0.007		0.16 (−0.33, 0.65)	0.539
			MR-PRESSO (O-C)	Estimate	10	**0.14 (0.03, 0.25)**	**0.010**	9	**0.38 (0.03, 0.72)**	**0.032**
			Weighted median	Estimate		0.15 (0.03, 0.26)	0.012		0.44 (0.03, 0.84)	0.036

*MR, Mendalian randomization; IVW, inverse-variance-weighted; SIMEX, simulation extrapolation; PRESSO (O-C), pleiotropy residual sum and outlier (outlier-correction); LDL, low-density lipoprotein; CAD, coronary artery disease; CI, confidence interval; FPG, fasting plasma glucose; Hb1Ac, hemoglobin A1c*.

**Table 6:** Significant results from bidirectional MR analysis.

					**Original study**			**Replication study**		
**No**.	**Exposure**	**Outcome**	**MR methods**	**Parameter**	***N***	**Estimate (95% CI)**	***P***	***N***	**Estimate (95% CI)**	***P***
1	CAD	FPG	IVW	Estimate	30	0.00 (−0.02, 0.02)	0.873	83	0.00 (−0.01, 0.01)	0.834
			MR Egger	Intercept		0.00 (−0.01, 0.01)	0.352		**0.00 (−0.01, 0.01)**	**0.813**
				Slope		−0.02 (−0.06, 0.02)	0.437		**0.00 (−0.02, 0.03)**	**0.906**
			MR Egger (SIMEX)	Intercept		0.00 (−0.01, 0.01)	0.435		0.00 (−0.01, 0.01)	0.907
				Slope		0.01 (−0.01, 0.02)	0.781		0.00 (−0.01, 0.01)	0.844
			MR-PRESSO (O-C)	Estimate	29	**0.00 (−0.02, 0.02)**	**0.877**		No outlier	—
			Weighted median	Estimate		0.01 (−0.01, 0.03)	0.186		0.00 (−0.01, 0.01)	0.834
2	LDL cholesterol	HbA1c	IVW	Estimate	74	0.02 (−0.01, 0.05)	0.202	4	**−0.01 (−0.04, 0.03)**	**0.681**
			MR Egger	Intercept		0.00 (−0.01, 0.01)	0.263		0.00 (−0.02, 0.02)	0.796
				Slope		−0.01 (−0.06, 0.05)	0.859		−0.02 (−0.13, 0.09)	0.719
			MR Egger (SIMEX)	Intercept		0.02 (−0.01, 0.00)	0.056		0.00 (−0.01, 0.01)	0.513
				Slope		0.02 (−0.01, 0.05)	0.158		−0.02 (−0.03, 0.01)	0.548
			MR-PRESSO (O-C)	Estimate	71	**0.02 (−0.01, 0.04)**	**0.234**		Not enough IVs	—
			Weighted median	Estimate		−0.01 (−0.03, 0.03)	0.911		−0.02 (−0.04, 0.02)	0.376

*MR, Mendalian randomization; N, number of instruments; IVW, inverse-variance-weighted; SIMEX, simulation extrapolation; PRESSO (O-C), pleiotropy residual sum and outlier (outlier-correction); LDL, low-density lipoprotein; CAD, coronary artery disease; CI, confidence interval; FPG, fasting plasma glucose; Hb1Ac, hemoglobin A1c*.

### T2D-Related Traits and Cancers

We considered FPG, 2h-PG, and HbA1c as exposure traits. No significant causal association was observed between FPG and lung (*P* = 0.721), ovarian (*P* = 0.632), pancreatic (*P* = 0.768), and thyroid (*P* = 0.612) cancer. For FPG, IVs for lung, ovarian, pancreatic, and thyroid cancer satisfied the IV assumptions (F statistics >10, *I*^2^ > 90; Q, *P* > 0.05), and IVW was selected for MR analyses (Table 3). A pleiotropic effect was observed in breast cancer through Q (*P* < 0.001), Q′ (*P* < 0.001) statistics, and the MR-PRESSO global test (*P* < 0.001), and MR-PRESSO did not yield significant outcomes (*P* = 0.364). The NOME assumption was violated in SC lung cancer (*I*^2^ < 90), and the MR-Egger (SIMEX) method was used. The MR-Egger (SIMEX) method yielded nominally significant (*P* < 0.05) causal effects (*P* = 0.032). Furthermore, when 2h-PG was considered an exposure trait, none of the causal association were significant for breast (*P* = 0.303), lung (*P* = 0.721), SC lung (*P* = 0.037), ovarian (*P* = 0.632), pancreatic (*P* = 0.768), and thyroid (*P* = 0.612) cancer. In IVs for all cancers, except for breast cancer, we found no weak instrument bias (*F* > 10) and no heterogeneity (*Q, P* > 0.05; *Q*′, *P* > 0.05; MR-PRESSO global test, *P* > 0.05), and the IVW method was used. However, IVs for breast cancer have a measurement error (*I*^2^ < 90), and the MR-Egger (SIMEX) method was used. Moreover, regarding HbA1c, no significant association was observed between HbA1c and breast (*P* = 0.922), lung (*P* = 0.173), SC lung (*P* = 0.115), ovarian (*P* = 0.719), pancreatic (*P* = 0.374), and thyroid (*P* = 0.417) cancer. Evidence of violations of IV assumptions for all cancers was obtained (*F* statistics >10; *Q, P* > 0.05; *Q*′, *P* > 0.05; MR-PRESSO global test, *P* > 0.05), and IVW was applied.

For lung, breast, and ovarian cancer, we assumed an OR of 1.2, and we determined the statistical power at between 40 and 70%. The highest power was observed for FPG with the highest *R*^2^, followed by HbA1c and 2h-PG. The estimated statistical power was the highest (>80%) for SC lung cancer for all T2D-related traits owing to a sample size of >100,000 individuals if the standardized effect size is assumed to be same. However, for pancreatic and thyroid cancers, the sample size was small (3,835 and 1,080, respectively), thus decreasing the statistical power, indicating the possibility of false-negative results. The overall estimated power (Figure 2) revealed no causal effect of FPG, 2h-PG, and HbA1c on breast, lung, SC lung, ovarian, pancreatic, and thyroid cancers (*P* < 0.008 after Bonferroni correction; Table 4).

### T2D-Related Traits and Vascular Diseases

With respect to vascular diseases, the data retrieved referred to a sample size of >100,000 patients, translated into a power ≥80%, except for the detection of an OR of 1.1. We found no causal effect of FPG, 2h-PG, or HbA1c on CKD, HDL levels, stroke, or stroke subtype; however, two significant causal relationships were observed between FPG and CAD and HbA1c with LDL level. Interestingly, three T2D-related traits used as criteria for the diagnosis of T2D showed different results for the same phenotype.

First, FPG showed no causal effects on CKD (*P* = 0.351) and cardioembolic stroke (*P* = 0.118), while it was nominally significant in the context of small-vessel stroke (*P* = 0.025). Nominally significant results were observed for CAD (*P* = 0.045), while nonsignificant results were observed for HDL cholesterol (*P* = 0.265), LDL cholesterol (*P* = 0.225), and stroke (*P* = 0.135). IVs for CKD and cardioembolic stroke strongly satisfied the IV assumptions (F statistics, >10; *I*^2^ > 90; *Q, P* > 0.05; *Q*′, *P* > 0.05; MR-PRESSO global test, *P* > 0.05), and the IVW approach was selected (Table 3). Of note, in the case of CAD, HDL/LDL cholesterol, and stroke, IVs were heterogeneous (*Q, P* < 0.05; *Q*′, *P* < 0.05; MR-PRESSO global test, *P* < 0.05); therefore, the MR-PRESSO method was applied. For small-vessel stroke, IVs were heterogeneous (*Q, P* < 0.05; *Q*′, *P* > 0.05; MR-PRESSO global test, *P* > 0.05); therefore, the MR-Egger method was applied.

Second, nonsignificant causal effects were observed for 2h-PG on CAD (*P* = 0.301), CKD (*P* = 0.183), stroke (*P* = 0.338), cardioembolic stroke (*P* = 0.530), small-vessel stroke (*P* = 0.084), HDL cholesterol (*P* = 0.074), and LDL cholesterol (*P* = 0.241). IVs for CKD, stroke, cardioembolic stroke, and small-vessel stroke strongly satisfied the IV assumptions (*F* statistics >10; *Q, P* > 0.05; *Q*′, *P* > 0.05; MR-PRESSO global test, *P* > 0.05), and the IVW method was used. On the other hand, because the measurement error (*I*^2^ < 90) of CAD and HDL/LDL cholesterol suggested heterogeneity (*Q, P* < 0.05; *Q*′, *P* < 0.05; MR-PRESSO global test, *P* < 0.05), the MR-Egger (SIMEX) method was used.

Third, no causal effects of HbA1c were observed on CKD (*P* = 0.337), HDL cholesterol (*P* = 0.206), and stroke (*P* = 0.567); conversely, there were nominally significant implications for cardioembolic stroke (*P* = 0.023) and small-vessel stroke (*P* = 0.046). IVs for CKD, HDL cholesterol, stroke, cardioembolic stroke, and small-vessel stroke strongly satisfied the IV assumptions (*F* statistics >10; *Q, P* > 0.05; *Q*′, *P* > 0.05; MR-PRESSO global test, *P* > 0.05), and the IVW method was once again selected. Owing to the heterogeneity in CAD and LDL cholesterol (*Q, P* < 0.05; *Q*′, *P* < 0.05; MR-PRESSO global test, *P* < 0.05), the MR-PRESSO method was again considered, and nominally significant results were obtained for LDL cholesterol (*P* = 0.010), while nonsignificant results were obtained for CAD (*P* = 0.069).

### Two Significant Causal Relationship

**Figure 3:**
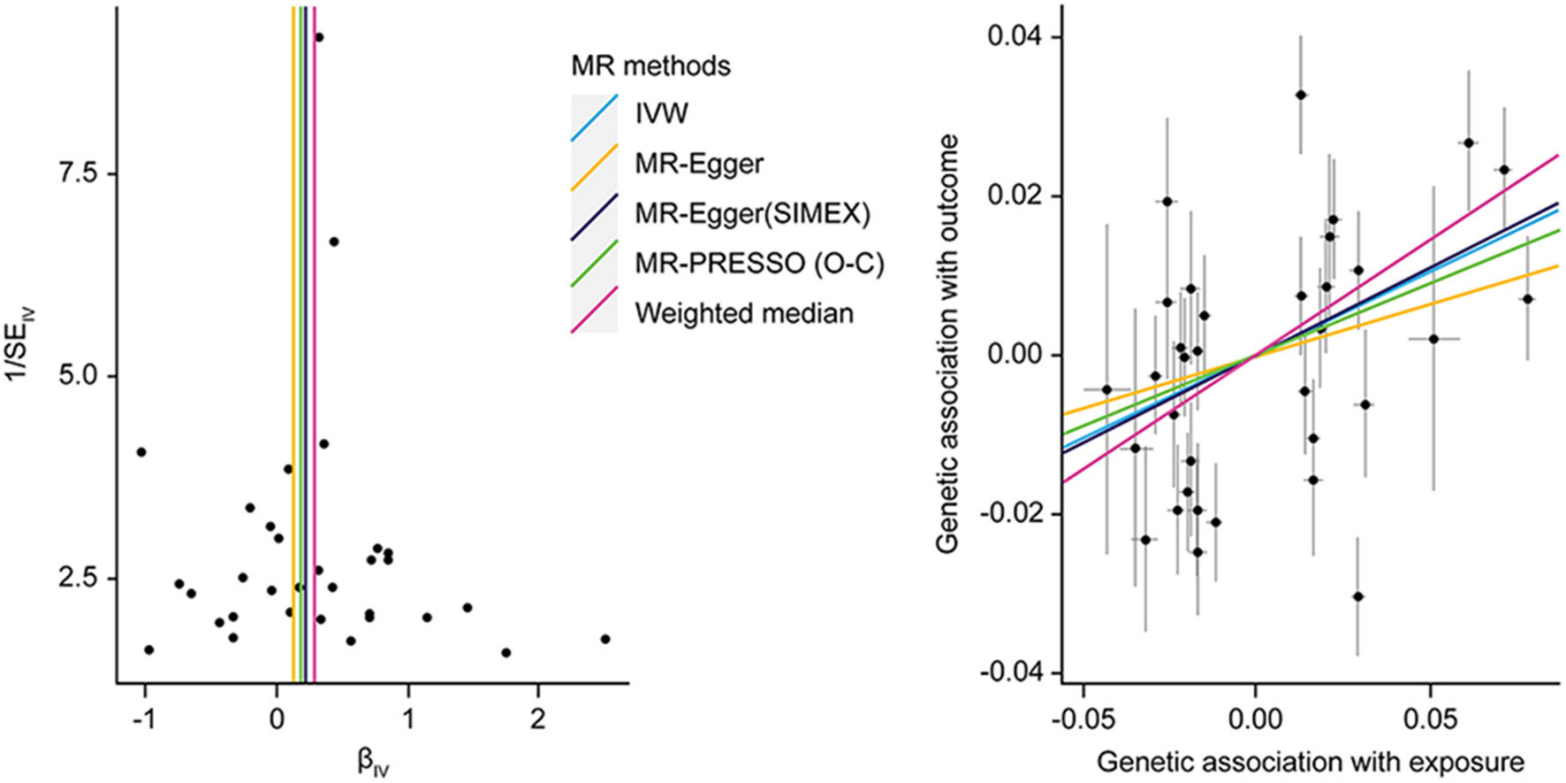
MR analysis of the effect of FPG on CAD. **(right)** Funnel plot displaying individual causal effect estimates for FPG on CAD. Dots representing the estimated causal effect for each IV. **(left)** The association between the effect size estimates on the FPG (*X*-axis) and CAD (*Y*-axis) for all SNPs that served as IVs. FPG, fasting plasma glucose; CAD, coronary artery disease; IV, instrumental variable; SNP, single-nucleotide polymorphism; SIMEX, simulation extrapolation; PRESSO (O-C), pleiotropy residual sum and outlier (outlier-correction).

Significant causal effects were found for FPG-CAD and HbA1c-LDL cholesterol. Regarding FPG-CAD, all SNP-exposure and SNP-outcome effects are presented in Supplementary Table 1. We found two SNPs correlating significantly with CAD (rs1260326: *P* = 2.40 × 10^−5^; rs7651090: *P* = 1.20 × 10^−5^); however, given that they exhibited balanced (nondirectional) pleiotropy, they were not excluded from the analysis (but were excluded from MR-PRESSO tests). A generated funnel plot showed symmetry, indicating heterogeneity due to directional horizontal pleiotropy (Figure 3A). The associations of the variants with FPG and CAD are shown in a scatter plot with five MR-fitted lines (Figure 3B). In the replication study using the same IVs and different GWAS data for outcome (PmID = 29,212,778, *N* = 296,525, *P* = European, and unit = logOR), there was no weak instrument bias of IVs (*N* = 34, *F* statistics = 43.5), but the heterogeneity assumption was violated (*Q, P* < 0.05; *Q*′, *P* < 0.05; MR-PRESSO global test, *P* < 0.05). Therefore, MR-PRESSO method was selected. Importantly, we found that FPG showed a positive causal effect on CAD (*P* = 0.002) (Table 5). Moreover, we verified that reverse causality did not exist. Upon bidirectional MR analysis in the original study, 29 SNPs were considered instrument variables. Weak instrument bias (*F* statistics, 77.4) and the NOME assumption (*I*^2^ = 92.6) were preserved; however, heterogeneity was observed (*Q, P* < 0.05; *Q*′, *P* < 0.05; MR-PRESSO global test, *P* < 0.05). The MR-PRESSO revealed no causal effect of CAD on FPG (*P* = 0.877) (Table 6). Upon bidirectional MR analysis in the replication study, 83 SNPs were considered instrument variables. Weak instrument bias (*F* statistics, 77.2) and the NOME assumption (*I*^2^ = 92.4) were preserved; however, heterogeneity was observed (*Q, P* < 0.05; *Q*′, *P* < 0.05; MR-PRESSO global test, *P* > 0.05). The MR-Egger revealed no causal effect of CAD on FPG (*P* = 0.906; Table 6).

**Figure 4:**
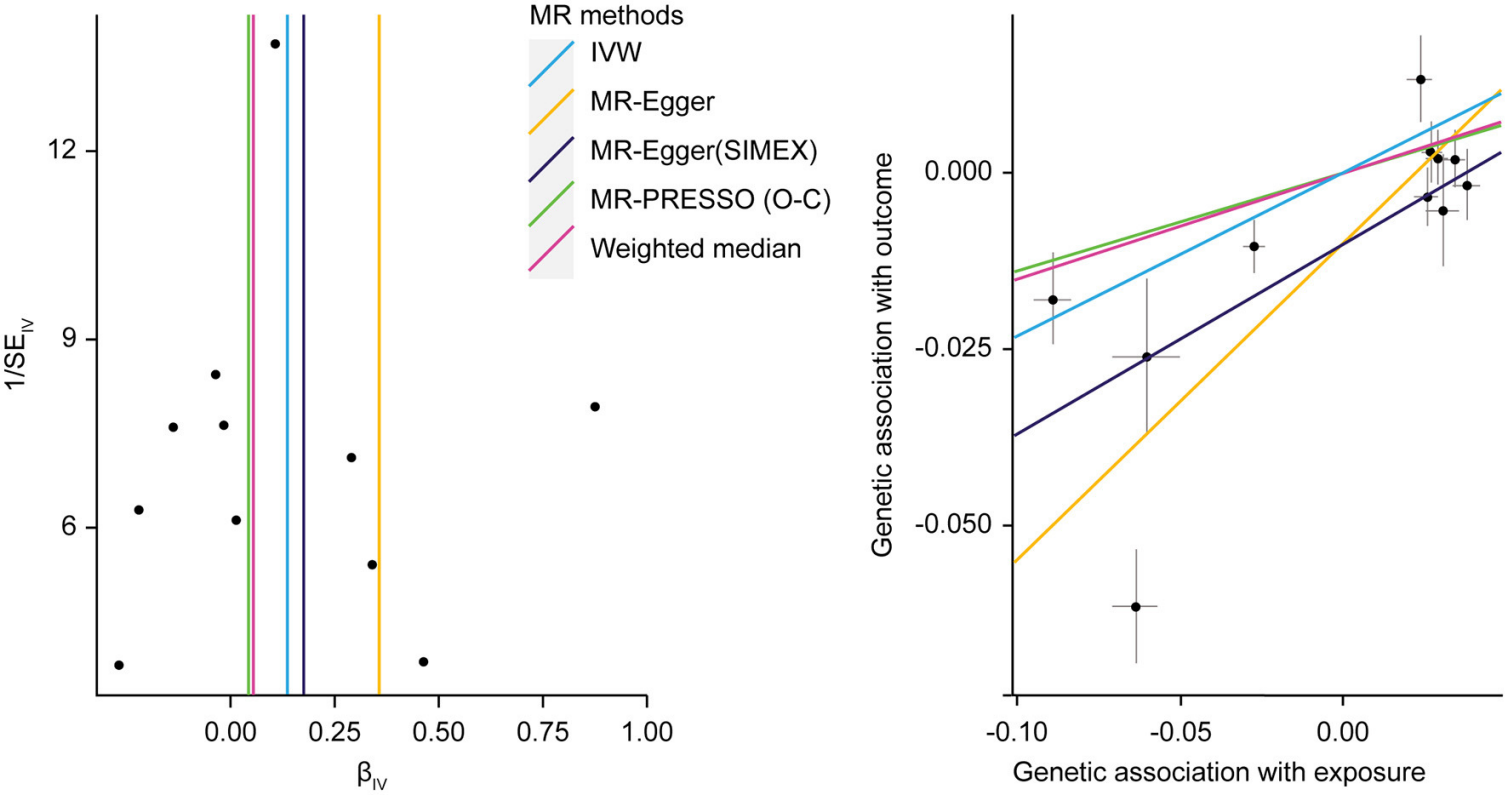
MR analysis of the effect of HbA1c on LDL levels. **(right)** Funnel plot displaying individual causal effect estimates for HbA1c on LDL levels. Dots represent the estimated causal effect for each IV. **(left)** The relationship between the effect size estimates on HbA1c (*X*-axis) and LDL level (*Y*-axis) for all SNPs that served as IVs. MR, Mendalian randomization; HbA1c, hemoglobin A1c; LDL, low-density lipoprotein; IV, instrumental variable; SNP, single-nucleotide polymorphism; SIMEX, simulation extrapolation; PRESSO (O-C), pleiotropy residual sum and outlier (outlier-correction).

Regarding HbA1c and LDL cholesterol, SNP-exposure and SNP-outcome effects (Supplementary Table 2) indicated that one SNP significantly correlated with the levels of LDL (rs1800562: *P* = 4.42 × 10^−4^) and was, therefore, excluded from the MR-PRESSO analysis. Figure 4A shows a funnel plot indicating slight nonsymmetry, suggesting the presence of heterogeneity due to directional horizontal pleiotropy. The scatter plot in Figure 4B shows the associations of the variants with HbA1c and LDL levels. Replication analysis using the same IVs and different GWAS data for the outcome-SNP effect (PmID = 28,887,542, *N* = 9,961, *P* = European, unit = mg/dl) revealed no evidence of a weak instrument bias (*N* = 11; *F* statistics, 77.6) and no heterogeneity (*Q, P* > 0.05; *Q*′, *P* > 0.05; MR-PRESSO global test > 0.05); however, the NOME assumption (*I*^2^ = 87.9) was violated. Therefore, the MR-PRESSO was used, revealing significant results for the causal effect of HbA1c on LDL cholesterol (*P* = 0.032; Table 5). Moreover, we verified that reverse causality did not exist. Upon bidirectional MR analysis in the original study, 74 SNPs were considered instruments, and no weak instrument bias was noted (*F* statistics, 153.9), with no violation of the NOME assumption (*I*^2^ = 97.7). However, heterogeneity was observed (*Q, P* < 0.05; *Q*′, *P* < 0.05; MR-PRESSO global test, *P* < 0.05), and the MR-PRESSO revealed no causal effect of LDL cholesterol on HbA1c (*P* = 0.234; Table 6). As per the bidirectional MR analysis for the replication study, four SNPs were considered instrument variables. No weak instrument bias (*F* statistics, 42.9) and no heterogeneity (*Q, P* > 0.05; *Q*′, *P* > 0.05; MR-PRESSO global test > 0.05) were observed; however, a violation of the NOME assumption (*I*^2^ = 4) was noted. Accordingly, the IVW method was considered, and no causal effect of LDL cholesterol on HbA1c was observed (*P* = 0.681; Table 6).

## Discussion

In this study, we performed MR analysis of the effect of T2D-related traits on 13 human health phenotypes using GWAS results and data from the MR-Base registry. In particular, MR analysis was conducted according to three T2D-related criteria (FPG and 2h-PG from the OGTT and HbA1c). MR analyses reduce potential confounding effects and reverse causation, and our results are concurrent with those of previous epidemiological studies. Previous large meta-analyses or systematic reviews of epidemiological studies show that the association between T2D and cancer development is unclear (Tsilidis et al., 2015). Moreover, most epidemiological studies report limitations in findings of T2D-related association with cancers because they were based on self-reported health assessments with high specificity (>90%) but low sensitivity (66%) as compared with medical records (Okura et al., 2004). Recently, results of MR analysis indicated no strong evidence supporting a causal relationship between T2D and major solid tumors (stomach, colorectal, liver, pancreas, lung, breast, and prostate) (Goto et al., 2020). Similarly, in the present study, analysis of European data from the MR-Base registry revealed no significant causal effect of T2D-related traits on breast, lung, SC lung, ovarian, pancreatic, and thyroid cancers. Although T2D and cancer share a number of risk factors, such as hyperglycemia, insulin resistance, and dyslipidemia, a relationship between the diseases has not been fully demonstrated (Vigneri et al., 2009). Additionally, studies have reported correlations between hypoglycemic agents and cancer incidence, although these findings remain controversial (Alimova et al., 2009; Currie et al., 2009).

In T2D patients, the risk of death from cardiovascular disease increases along with elevated FPG and HbA1c levels, with HbA1c level correlated with microvascular and microvascular complications (Kannel and McGee, 1979; Group, 1998; Okura et al., 2004). Therefore, hyperglycemia represents a strong independent factor for cardiovascular disease, with the risk increasing 2–3-fold in men and 3–4-fold women diagnosed with T2D relative to those without T2D (Kannel and McGee, 1979; Okura et al., 2004). A longitudinal study involving follow-up for 8 years of 2,363 nondiabetic adults between the ages of 50 and 75 years reported significant association between 2h-PG and HbA1c levels and an increased risk of death from cardiovascular disease (De Vegt et al., 1999). Moreover, that study identified HbA1c level as not only predictive of improved better mortality from cardiovascular disease relative to FPG and 2h-PG (Park et al., 1996) but also an independent risk factor for atherosclerosis and cardiovascular disease independent of T2D (Nakamura et al., 1993; Kanauchi et al., 2001). In the present study, our findings indicated that vascular disease and LDL level were significantly linked with HbA1c level but not FPG or 2h-PG.

We found that different characteristics related to FPG, 2h-PG, and HbA1c differentially influenced IV characteristics. The 2h-PG results from an OGTT represent a standard test for T2D diagnosis. Although 2h-PG testing is more highly sensitive and specific than FPG testing, its low reproducibility is a disadvantage (Peters et al., 1996). The low reproducibility is a consequence of changes in 2-h glucose concentrations for each measurement within a 48-h or 1-week time period in the same individual. On the other hand, FPG testing is simple and reproducible; however, the sensitivity for T2D diagnosis is poor because it does not allow accurate identification of hyperglycemia after glucose load (Davidson et al., 1999). HbA1c reflects overall tissue protein glycation and can better reflect the overall biological effect of blood sugar as a 3-month average blood sugar estimate (Peterson et al., 1998); however, HbA1c measurements can be affected by hemoglobin disease, chronic renal failure, testing methods, and/or specific dosage (Barr et al., 2002). Therefore, these findings suggest that the measurement error associated with SNP-exposure associations might be large when using any of these criteria. A previous study showed that calculation of the *I*^2^ value confirmed the inadequacy of the NOME assumption due to measurement error related to 2h-PG testing (Bowden et al., 2016b). Furthermore, reports indicated that the HbA1c level shows less variability in day-to-day within-person variance than FPG (<2% for HbA1c vs. 12–15% for FPG) (Ollerton et al., 1999), and the intraindividual coefficient of variation for FPG (6.4%) is less than that for 2h-PG (16.7%) (Mooy et al., 1996). Therefore, MR analysis using 2h-PG as an exposure can be expected to increase the reliability of MR-Egger (SIMEX) findings relative to other methods. In the cases of FPG and HbA1c, IVW results and the sensitivity analysis methods should be examined more broadly.

We performed MR analysis using public data from previous large-scale GWAS studies. Producing in-house genetic data is expensive and requires substantial human resources, making it difficult for many individual researchers lacking access to appropriate datasets. A two-sample MR approach represents an effective method for discovering novel causal relationships through the use of available large-scale GWAS datasets. Additionally, MR analysis excludes confounding effects by using SNPs associated with exposure as genetic instruments, which also reduces the adverse effects of inaccurate data on hindering identification of relationships between exposure and outcome. Furthermore, since the instrument strength is not significantly affected by the number of IVs (Burgess et al., 2011), even if the number of the instruments are small, it can be used as a useful IVs if the effect size of association is strong.

The present MR analysis has several limitations. First, some subjects may have overlapped between the two data sets with respect to the estimates of instrument exposure and instrument outcome, which could lead to inflated type 1 error rates and false-positive findings (Burgess et al., 2016). Furthermore, MR analyses are based on the GWAS. GWAS requires numerous subjects, often in multiple cohorts. Disease definition can differ among different cohorts. Third, we mostly included studies involving a predominantly European population with few individuals of other ancestries (mixed); hence, the present results may not be applicable to other racial backgrounds. Finally, if GWAS summary results for a mixed population is used instead of homogeneous group of individuals, such as those of European ancestry in MR analysis, the result can be confounded by population stratification. Nevertheless, our MR study not only validated the results of previous epidemiology studies but also suggested the difference among FPG, 2h-PG, and HbA1c for the major clinical outcomes. We hope that it supports various studies based on the laboratory markers in T2D.

## Data Availability Statement

All datasets generated for this study are included in the Supplementary Material and available at https://www.mrbase.org/ and https://gwas.mrcieu.ac.uk/.

## Ethics Statement

All datasets used are publicly available. Ethical review and informed consent had been obtained in all of the original studies.

## Author Contributions

HJ analyzed and interpreted the results and wrote the manuscript. SW and SL designed the study. All authors revised this paper critically for important intellectual content. All authors contributed to the article and approved the submitted version.

## Conflict of Interest

SW was employed by the company, RexSoft. The remaining authors declare that the research was conducted in the absence of any commercial or financial relationships that could be construed as a potential conflict of interest.
