# Supplementary material for: Causal Evaluation of Laboratory Markers in Type 2 Diabetes on Cancer and Vascular Diseases Using Various Mendelian Randomization Tools
Source: Front Genet. 2020 Dec 21;11:597420. doi: 10.3389/fgene.2020.597420 (PMC7780896; doi:10.3389/fgene.2020.597420)
Supplement: Supplementary file 1 [file Data_Sheet_1.PDF]

Supplementary Material

Supplementary Table 1. Instrument variables for FPG and CAD.

Chr	SNP	EA	OA	SNP Exposure			SNP Outcome		
				Beta	SE	*P*	Beta	SE	*P*
1	rs17712208	A	T	0.051	0.007	3.22×10^{-12}	0.002	0.019	0.920
2	rs1260326	C	T	0.029	0.002	2.17×10^{-41}	−0.030	0.007	2.4×10^{-5}
2	rs479661	A	G	−0.019	0.003	8.56×10^{-12}	−0.014	0.009	0.150
2	rs560887	C	T	0.071	0.003	1.40×10^{-178}	0.023	0.008	0.002
3	rs11708067	G	A	−0.023	0.003	1.30×10^{-18}	−0.020	0.008	0.017
3	rs11715915	T	C	−0.012	0.002	4.90×10^{-08}	−0.021	0.008	0.005
3	rs1280	C	T	−0.026	0.003	8.56×10^{-18}	0.007	0.010	0.520
3	rs7651090	G	A	0.013	0.002	1.75×10^{-08}	0.033	0.007	1.2×10^{-5}
5	rs4869272	T	C	0.018	0.002	1.02×10^{-15}	0.003	0.007	0.660
6	rs9368222	A	C	0.014	0.002	1.00×10^{-09}	−0.005	0.008	0.560
7	rs17168486	T	C	0.031	0.003	3.17×10^{-28}	−0.006	0.009	0.500
7	rs2191349	T	G	0.029	0.002	1.28×10^{-42}	0.011	0.007	0.130
7	rs6943153	C	T	−0.015	0.002	1.63×10^{-12}	0.005	0.007	0.500
7	rs6975024	C	T	0.061	0.003	2.88×10^{-99}	0.027	0.009	0.003
7	rs882020	T	C	0.021	0.003	3.04×10^{-12}	0.015	0.010	0.150
8	rs11558471	G	A	−0.029	0.002	7.80×10^{-37}	−0.003	0.008	0.709
8	rs983309	G	T	−0.026	0.003	6.29×10^{-15}	0.019	0.011	0.072
9	rs10811661	C	T	−0.024	0.003	5.65×10^{-18}	−0.008	0.009	0.410
9	rs10814916	C	A	0.016	0.002	2.26×10^{-13}	−0.010	0.007	0.130
9	rs16913693	G	T	−0.043	0.007	3.51×10^{-11}	−0.004	0.021	0.830
9	rs3829109	A	G	−0.017	0.003	1.13×10^{-10}	−0.025	0.008	0.002
10	rs11195502	T	C	−0.032	0.004	1.97×10^{-18}	−0.023	0.012	0.048
10	rs7903146	T	C	0.022	0.002	2.71×10^{-20}	0.017	0.008	0.027
11	rs10830963	G	C	0.078	0.003	1.00×10^{-200}	0.007	0.008	0.370
11	rs11603334	A	G	−0.019	0.003	1.12×10^{-11}	0.008	0.010	0.390
11	rs11607883	A	G	−0.021	0.002	6.32×10^{-24}	0.000	0.007	0.960
11	rs174576	A	C	−0.020	0.002	1.18×10^{-18}	−0.017	0.007	0.019
11	rs749067	C	T	−0.017	0.002	6.12×10^{-15}	0.001	0.007	0.940
12	rs10747083	A	G	0.013	0.002	7.57×10^{-09}	0.007	0.008	0.320
13	rs11619319	G	A	0.020	0.002	1.33×10^{-15}	0.008	0.008	0.320
14	rs3783347	T	G	−0.017	0.003	1.32×10^{-10}	−0.020	0.008	0.020
15	rs4502156	C	T	−0.022	0.002	1.38×10^{-25}	0.001	0.007	0.890
20	rs6072275	A	G	0.016	0.003	1.66×10^{-08}	−0.016	0.010	0.110
20	rs6113722	A	G	−0.035	0.005	2.49×10^{-11}	−0.012	0.018	0.500

FPG, fasting plasma glucose; CAD, coronary artery disease; Chr, chromosome; EA, effect allele; OA, other all ele; SE, standard error; LDL, low−density lipoprotein; HbA1, hemoglobin A1c; SNP, single-nucleotide polym orphism.

Supplementary Table 2. Instrument variables for HbA1c and LDL cholesterol.

Chr	SNP	EA	OA	SNP Exposure			SNP Outcome		
				Beta	SE	*P*	Beta	SE	*P*
1	rs2779116	T	C	0.024	0.004	2.75×10^{-09}	0.013	0.006	0.106
2	rs552976	G	A	0.029	0.003	8.16×10^{-18}	0.002	0.004	0.424
6	rs1800562	A	G	−0.064	0.007	2.59×10^{-20}	−0.062	0.008	4.42×10^{-04}
7	rs1799884	T	C	0.038	0.004	1.45×10^{-20}	−0.002	0.005	0.988
8	rs4737009	A	G	0.027	0.004	6.12×10^{-12}	0.003	0.004	0.574
8	rs6474359	C	T	−0.060	0.011	1.18×10^{-08}	−0.026	0.011	0.030
10	rs16926246	T	C	−0.089	0.006	3.11×10^{-54}	−0.018	0.007	0.001
11	rs1387153	T	C	0.026	0.004	3.96×10^{-11}	−0.003	0.004	0.490
13	rs7998202	G	A	0.031	0.005	5.24×10^{-09}	−0.005	0.008	0.804
17	rs1046896	T	C	0.035	0.003	1.58×10^{-26}	0.002	0.004	0.528
22	rs855791	G	A	−0.027	0.004	2.74×10^{-14}	−0.010	0.004	0.003

Chr, chromosome; EA, effect allele; OA, other allele; SE, standard error; LDL, low−density lipoprotein; HbA1 c, hemoglobin A1c; SNP, single-nucleotide polymorphism.
